# Mindfulness Affects the Level of Psychological Distress in Patients With Lung Cancer *via* Illness Perception and Perceived Stress: A Cross-Sectional Survey Study

**DOI:** 10.3389/fpsyg.2022.857659

**Published:** 2022-04-07

**Authors:** Xu Tian, Ling Tang, Li-Juan Yi, Xiao-Pei Qin, Gui-Hua Chen, Maria F. Jiménez-Herrera

**Affiliations:** ^1^Department of Nursing, Universitat Rovira i Virgili, Tarragona, Spain; ^2^Chongqing University Cancer Hospital, Chongqing, China; ^3^Department of Rehabilitation Medicine, Jiaozuo People’s Hospital, Jiaozuo, China; ^4^Department of Nursing, The Second Affiliated Hospital of Chongqing Medical University, Chongqing, China

**Keywords:** lung cancer, psychological distress, mindfulness, illness perception, perceived stress, structural equation model

## Abstract

**Purpose:**

The aims of the study were first to investigate the association between illness perception and psychological distress and second to determine whether mindfulness affects psychological distress *via* illness perception and perceived stress in patients with lung cancer.

**Methods:**

Among 300 patients with lung cancer who participated in this cross-sectional study, 295 patients made valid responses to distress thermometer (DT), the Five Facet Mindfulness Questionnaire (FFMQ), the Brief Illness Perception Questionnaire (B-IPQ), and the Perceived Stress Scale (PSS) between January and July 2021. The possible pathways of mindfulness affecting psychological distress were analyzed based on the structural equation modeling analysis.

**Results:**

A total of 24.4% patients with lung cancer had DT > 4. Illness perception (β = 0.17, *p* = 0.002) and perceived stress (β = 0.23, *p* < 0.001) had a direct effect on psychological distress. Mindfulness had a direct effect on illness perception (β = −0.16, *p* = 0.006) and mindfulness indirectly influenced psychological distress (β = −0.04, *p* = 0.009) through affecting illness perception alone or simultaneously affecting both the illness perception and perceived stress in patients with lung cancer.

**Conclusion:**

Lung cancer suffered from varying levels of psychological distress. Mindfulness may alleviate psychological distress by reducing the level of illness perception and perceived stress. We suggest developing a comprehensive factor model to clarify potential mechanisms of mindfulness on psychological distress due to the very low effect of mindfulness on psychological distress *via* illness perception and perceived stress.

## Introduction

According to the National Comprehensive Cancer Network (NCCN) guideline, psychological distress is a multifactorial and unpleasant emotional experience, involving changes in psychological, social, spiritual, and physical aspects (Riba et al., 2019). Psychological distress can be detected at any stage in patients with cancer and even remain throughout the cancer trajectory (Gao et al., 2010). Overall, studies reported a prevalence of 20–52% for psychological distress [distress thermometer (DT) > 4] among patients with cancer (Funk et al., 2016; Mehnert et al., 2018). However, compared to patients with other types of cancers, patients with lung cancer reported the highest incidence of psychological distress (Zabora et al., 2001), with a detection rate of 17.0–73.0% (Lynch et al., 2010; Chambers et al., 2015; Tian et al., 2021a). It is noted that approximately 220 million new lung cancer cases were estimated in 2020, ranking second place among all the cancers (Sung et al., 2021). Therefore, the anticipated prevalence of psychological distress among patients with lung cancer should be especially emphasized.

Psychological distress has become a major psychological problem faced by patients with cancer because it has been demonstrated to be associated with the occurrence of several adverse consequences (Riba et al., 2019). As an example, psychological distress was found to be the contributor to the interruption of anticancer treatment (Mausbach et al., 2015; Lin et al., 2017; Yee et al., 2017). Meanwhile, psychological distress has also been found to be associated with longer hospital stays (Nipp et al., 2017), poor quality of life (Chambers et al., 2015), and increased risk of mortality (Batty et al., 2017; Hamer et al., 2009). Moreover, psychological distress was evidenced to accelerate the growth of tumor cells (Zhang et al., 2020a). Therefore, to develop effective intervention protocols to address psychological distress among patients with lung cancer, it is critically important to clarify the potential mechanisms involved in the development and progress of psychological distress (Riba et al., 2019).

## Background

Mindfulness refers to an individual’s focused attention on the present moment and non-judgmental awareness (Kabat-Zinn, 2003). As a protective source of negative psychological outcomes, several studies have suggested the positive effects of mindfulness among different populations (Kashiwazaki et al., 2020), even in the general population (Freudenthaler et al., 2017). At present, several meta-analyses have demonstrated that interventions involving mindfulness elements (Cillessen et al., 2019; Zhang et al., 2019; Nnate et al., 2021; Rieger et al., 2021), such as mindfulness-based cognitive therapy (MCT), mindfulness-based art intervention, and mindfulness-based stress reduction (MBSR), significantly improved the psychological wellbeing of patients with cancer. It should be noted that, moreover, our previous study consistently determined the negative association between mindfulness and psychological distress among patients with lung cancer and further clarified the influence of mindfulness on psychological distress through the mediating role of social support and perceived stigma, with a slight total effect of 0.048 (Lei et al., 2021). However, are there other potential mechanisms else in the association between mindfulness and psychological distress among patients with lung cancer to be discovered and elucidated?

Illness perception refers to an individual’s reflection in both the cognitive and emotional aspects and coping styles through personal knowledge and experiences when one confronts symptoms or illness threats, which have been revealed to have an impact on health outcomes (Leventhal et al., 2016). In a prospective, longitudinal, and observational study, illness perceptions were demonstrated as a potential predictor of psychological distress in patients with non-muscle-invasive bladder cancer (Zhang et al., 2020b). Meanwhile, illness perception has been found to predict psychological distress in head and neck cancer survivors (Zhang et al., 2018), esophageal cancer survivors (Dempster et al., 2011), and breast cancer survivors (Zhang et al., 2017). However, it is not yet known whether the same association holds for patients with lung cancer. Moreover, no study has investigated the association between mindfulness and illness perception; however, mindfulness-based interventions have been found to reduce the level of negative illness perceptions in patients with rheumatoid arthritis (Dalili and Bayazi, 2019) or acute coronary syndrome (Nasiri et al., 2020). We, therefore, assume that the association between mindfulness and illness perception also holds among patients with lung cancer.

Perceived stress refers to an individual’s subjective perception of stress and assessment of the ability of processing stress (Kim and Jang, 2020), which was positively related to psychological distress among patients with lung cancer in our previous study (Tian et al., 2021a). Meanwhile, perceived stress was also speculated to be associated with mindfulness because mindfulness-based intervention protocols had generally been shown to reduce stress (Lengacher et al., 2021). Interestingly, the negative association between mindfulness and perceived stress has been detected in patients with digestive tract cancer (Zhong et al., 2019). Moreover, some studies have also investigated the relationship between illness perception and perceived stress and found that individuals will experience greater levels of perceived stress if they negatively perceived their illness (Miceli et al., 2019). Unfortunately, these relationships of variables introduced above have not yet been determined in patients with lung cancer.

In the light of the above, we performed this study to examine three hypotheses as follow: (a) illness perception is positively associated with perceived stress and psychological distress, (b) mindfulness can influence psychological distress through illness perception, and (c) mindfulness has an impact on psychological distress through simultaneously influencing illness perception and perceived stress among patients with lung cancer.

## Methods

### Study Design

This study was a cross-sectional descriptive survey design.

### Participants

We recruited eligible patients with convenience sampling method from a tertiary hospital in Chongqing between January and July 2021 according to the inclusion criteria which was designed according to our previous studies (Tian et al., 2021a,b): adult patients were diagnosed with lung cancer based on definitive and route methods and confirmed to have ability to clearly and accurately read and write. Patients who were confirmed to have a mental disorder or received psychological treatment before eligibility evaluation or participated in those studies with similar study aims were excluded from this study.

### Sample Size

In this study, we used structural equation modeling technique with maximum likelihood to examine all the paths between variables, the *N*:*q* rule with a ratio of 10/1 was, therefore, used to calculate the theoretical sample size, in which *N* and *q* indicate required cases and the number of parameters that require statistical estimates, respectively (McDonald and Ho, 2002). In this study, *q* was identified to be 10 and, thus, a minimum sample size of 120 was calculated under the consideration of 20% invalid questionnaires.

### Study Variables

Demographic information was collected using a self-designed questionnaire and other variables, namely, psychological distress, illness perception, and perceived stress were measured using validated instruments, which have been translated into Chinese and published publicly in academic journals.

#### Demographic Information

In this study, we collected the following sociodemographic and clinical variables by the self-designed information collection form, namely, gender, age, educational degree, marital status, place of residence, occupational status, family history, pain, cancer metastasis, and tumor’s TNM stage.

#### Psychological Distress

We used DT to measure psychological distress at an 11-point thermometer scale from 0 to 10 in this study and 0 and 10 indicate no distress and extreme distress, respectively (Riba et al., 2019). The reliability and validity of DT have been extensively tested across different settings (Hong et al., 2015). According to several empirical studies, an individual with a score of 4 was defined to have clinically significant psychological distress (Donovan et al., 2014; Hong et al., 2015). There was no exception in China, a score of 4 was also demonstrated as the cutoff value of defining clinically significant psychological distress in Chinese cancer populations (Hong et al., 2015).

#### Mindfulness

We used the Five Facet Mindfulness Questionnaire (FFMQ), which was developed by Baer and colleagues in 2006 (Baer et al., 2006), to measure the level of mindfulness at a 5-point Likert scale. In the original version, total of 39 items were effectively pooled to assess mindfulness from five facets as follows: observing, describing, acting with awareness, non-judging, and non-reacting (Baer et al., 2006). The original FFMQ has been translated into Chinese by Deng et al. (2011), with acceptable psychometric propertiesDeng et al., 2011.

#### Illness Perception

We used the Brief Illness Perception Questionnaire (B-IPQ), which was developed by Weinman et al. (1996), to measure emotional and cognitive representations of illness at an 8-item continuous linear scale from 0 to 10. Higher scores represent more negative illness perceptions. Broadbent et al. (2006) have shown the B-IPQ to have good test-retest reliability and predictive and discriminant validityBroadbent et al., 2006. The B-IPQ has been translated into Chinese (Xue and Lin, 0000) and has been widely used as a screening tool for assessing illness perceptions in China (Broadbent et al., 2006).

#### Perceived Stress

We used the 10-item Perceived Stress Scale (PSS), which was developed by Cohen et al. (1983), to measure the level of perceived stress at a 5-point Likert scale from 0 to 4. A higher score represents a greater stress level. The Cronbach’s alpha was 0.84 at the instrument development stage. The Chinese version of the 10-item PSS has been found to have the Cronbach’s alpha of 0.619 (Yuan and Lin, 2009).

### Procedure

We strictly performed this study following the provision of the Declaration of Helsinki. The Institutional Review Board (IRB) approved our protocol and assigned an ethical identifier of CZLS2021183-A to this protocol before enrollment commenced. Before conducting the formal survey, all eligible patients were informed about objectives and the risks and benefits of the study and required to sign informed consent. Study questionnaires were independently and anonymously completed by patients. The Strengthening the Reporting of Observational Studies in Epidemiology (STROBE) guidelines were utilized to guide us to report all the data (von Elm et al., 2014).

### Statistical Analysis

All the valid questionnaires were completely written by responders. Descriptive statistics for all the variables were calculated using Statistical Package for the Social Sciences (SPSS) version 22.0 (Chicago, IL, United States). Age, the score of psychological distress, mindfulness, social support, and perceived stigma were expressed as median with interquartile range (IQR) because all did not follow normal distribution according to the results from the Kolmogorov–Smirnov test. The Spearman’s rank correlation analysis was conducted using SPSS version 22.0 to examine the relations between mindfulness, illness perception, perceived stress, and psychological distress. The mediation model was tested using AMOS version 21.0 (Chicago, IL, United States). In these analyses, we used 2,000 bootstrap resamples and focused on the bias-corrected and accelerated CI. The following indices were calculated to evaluate the fitness of the overall model: the ratio of the chi-squared (χ^2^) to degrees of freedom (*df*), comparative fit index (CFI), goodness-of-fit index (GFI), adjusted GFI (AGFI), Tucker–Lewis index (TLI), incremental fit index (IFI), and root mean square error of approximation (RMSEA) with 90% CI. Model fit was regarded as good when a ratio of χ^2^/*df* was equal to or less than 3. For GFI and AGFI, a value of *p* < 0.90 indicates a good model fit. Moreover, CFI of ≥ 0.90 and RMSEA of < 0.05 were also suggesting a good model fit. A *p* < 0.05 indicated significance for all the analyses.

## Results

### Sample Characteristics

A total of 300 eligible patients with lung cancer were recruited to participate in this survey study eventually, of which 295 patients returned valid questionnaires, representing a valid response rate of 98.3%. Among these 295 patients, 72 patients were defined to have clinically significant psychological distress, with a detection rate of 24.4%. Sociodemographic and clinical variables of 295 patients are shown in Table 1. Most patients were men (71.2%) and did not get adequate education (67.1%) and a significant number of patients were married (97.6%). Most patients lived in urban (71.5%) and balanced medical expenditure with medical insurance (96.9%), and more than half of them suffered from cancer metastasis (64.1%). Although most patients had no family history (92.2%), a significant number of patients were at the advanced stage (81.0%) and had no or mild pain (99.0%).

**TABLE 1:** Psychological distress among patients with different sample characteristics (*N* = 295).

Characteristics	Frequency (%)	Mean rank	*Z/*χ*^2^*	*P*
Gender			−1.197	0.231^a^
Male	210 (71.2)	144.65		
Female	85 (28.8)	156.26		
Age, years			0.567	0.753^b^
18–40	4 (1.4)	164.00		
41–60	144 (48.8)	150.55		
>60	147 (49.8)	145.06		
Education			1.650	0.648^b^
Illiterate or elementary school	88 (29.8)	142.61		
Junior school	110 (37.3)	146.97		
Senior high school	57 (19.3)	149.27		
College or above	40 (13.6)	160.88		
Marital status			2.535	0.282^b^
Married	288 (97.6)	146.91		
Unmarried	1 (0.4)	199.50		
Widowed or divorced	6 (2.0)	191.67		
Residence			−0.120	0.905^a^
Urban	211 (71.5)	148.33		
Rural	84 (28.5)	147.17		
Occupation status			3.616	0.164^b^
Unemployed	112 (38.0)	145.60		
Employed	40 (13.6)	169.01		
Retired	143 (48.4)	144.00		
Medical insurance			0.502	0.478^a^
Self-paying	9 (3.1)	130.44		
Medicare	286 (96.9)	148.55		
Family history			−1.860	0.063^a^
No	272 (92.2)	150.38		
Yes	23 (7.8)	119.89		
Pain			27.307	<0.001^b^
No	190 (64.4)	131.84		
Mild	102 (34.6)	176.74		
Moderate	2 (0.7)	143.75		
Severe	1 (0.3)	295.00		
Cancer metastasis			−3.181	0.001^a^
No	106 (35.9)	166.66		
Yes	189 (64.1)	137.53		
Tumor stage			7.687	0.053^b^
I	30 (10.2)	159.30		
II	26 (8.8)	183.69		
III	38 (12.9)	141.16		
IV	201 (68.1)	142.99		

*^a^Mann–Whitney U test.*

*^b^Kruskal–Wallis H test.*

### Relationships of Psychological Distress, Mindfulness, Illness Perception, and Perceived Stress

Overall, the median score of psychological distress was 0 with an IQR of from 0 to 3. The score of mindfulness, illness perception, and perceived stress was 115 (109 to 119), 43 (39 to 47), and 20 (17 to 23), respectively. We designed Table 2 to display the relationships of psychological distress, mindfulness, illness perception, and perceived stress. The results of the Spearman’s rank correlation analyses suggested that all the variables were significantly correlated with one another.

**TABLE 2:** Spearman correlations for mindfulness, illness perception, perceived stress, and psychological distress.

Variable	Median (P_25_, P_75_)	Psychological distress	Mindfulness	Illness perception	Perceived stress
Psychological distress	0 (0, 3)	1			
Mindfulness	115 (109, 119)	−0.143*	1		
Illness perception	43 (39, 47)	0.233**	−0.181**	1	
Perceived stress	20 (17, 23)	0.235**	−0.116*	0.143*	1

*P < 0.05, **P < 0.01.

### Structural Equation Modeling of the Association of Psychological Distress, Mindfulness, Illness Perception, and Perceived Stress

**FIGURE 1:**
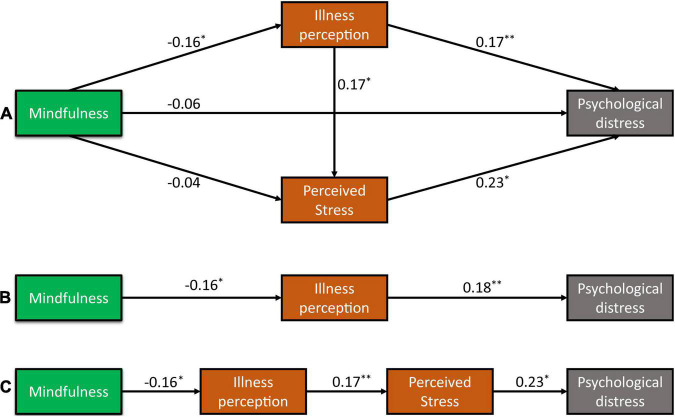
Mechanisms of mindfulness on psychological distress among Chinese patients with lung cancer. Theoretical mechanisms of mindfulness on psychological distress according to correlation analysis **(A)**, influencing of mindfulness on psychological distress through illness perception **(B)**, and influencing of psychological distress on psychological distress through the chain mediating role of illness perception and perceived stress **(C)**. *P < 0.05, **P < 0.01.

We first constructed the relationship structure of all variables according to the results of the correlation matrix. After conducting model fit analysis, we found that the direct path from mindfulness to psychological distress or perceived stress was not statistically significant (Figure 1A). We therefore eliminated those two paths to good fit the structural model (χ^2^/*df* = 0.867, CFI = 0.999, GFI = 1.000, CFI = 1.000, TLI = 1.019, and RMSEA = 0.000 [0.000 to 0.111]).

As illustrated in Figure 1A, illness perception (β = 0.18 and *p* = 0.002) and perceived stress (β = 0.23 and *p* < 0.001) had direct positive effects on psychological distress. The direct pathways from mindfulness to illness perception (β = −0.16 and *p* = 0.006) and from illness perception to perceived stress (β = 0.17 and *p* = 0.003) were all statistically significant.

The results from the bootstrap test for the significance of all pathways are shown in Table 3. Results for indirect pathways indicated that the indirect pathways between illness perception and psychological distress through perceived stress were statistically significant (*B* = 0.04, 95% CI [0.02–0.07], and *p* = 0.009). Overall, the total effect of illness perception on psychological distress was 0.22 [95% CI (0.12–0.30) and *p* = 0.007]. Furthermore, mindfulness had only an indirect negative effect on perceived stress through illness perception, with an indirect effect of −0.03 [95% CI (−0.07 to −0.01) and *p* = 0.009]. However, mindfulness had an impact on psychological distress through influencing only illness perception (Figure 1B) or influencing simultaneously illness perception and perceived stress (Figure 1C). Specifically, the indirect effect of mindfulness on psychological distress was −0.03 through mediating effect of illness perception alone, and the indirect effect of mindfulness on psychological distress was −0.01 through the chain mediating effect of illness perception and perceived stress. Overall, the total effect of mindfulness on psychological distress was −0.04 through two indirect pathways. The results suggested that illness perception and perceived stress play a chain mediating role in the relationship between mindfulness and psychological distress among patients with lung cancer.

**TABLE 3:** Effect estimates of mindfulness to psychological distress through illness perception and perceived stress.

	Direct effect (95% CI)	Indirect effect (95% CI)	Total effect (95% CI)
**Direct path**			
Mindfulness → Illness perception	−0.16 (−0.27, −0.07)	n.a.	−0.16 (−0.27, 0.07)
Illness perception → Perceived stress	0.17 (0.08, 0.26)	n.a.	0.17 (0.08, 0.26)
Illness perception → Psychological distress	0.18 (0.09, 0.26)	n.a.	0.18 (0.09, 0.26)
Perceived stress → Psychological distress	0.23 (0.10, 0.30)	n.a.	0.23 (0.10, 0.30)
Indirect path			
Illness perception → Psychological distress	0.18 (0.09, 0.26)	0.04 (0.02, 0.07)	0.22 (0.12, 0.30)
Mindfulness → Perceived stress	n.a.	−0.03 (−0.07, −0.01)	−0.03 (−0.07, −0.01)
Mindfulness → Psychological distress	n.a.	−0.04 (−0.07, −0.02)	−0.04 (−0.07, −0.02)

*CI, confidence interval; n.a., not available.*

## Discussion

Psychological distress has been demonstrated to be associated with several negative clinical outcomes such as interruption of anticancer treatment, poor quality of life, and higher morbidity and mortality (Riba et al., 2019). It is imperative to clarify the potential mechanisms of the development and progress of psychological distress among patients with lung cancer to develop a more effective intervention protocol (Lei et al., 2021; Riba et al., 2019). The major aim of this study is to determine whether negative illness perception is positively related to be psychological distress and whether mindfulness may have a protective effect on psychological distress through negatively influencing illness perception and perceived stress among patients with lung cancer.

After completing this study, we found that 24.4% of patients experienced clinically significant psychological distress, which was consistent with previous findings (Tian et al., 2021a) although there also are some studies that reported a higher detection rate (Hong et al., 2015; Carlson et al., 2004). It is possible that the relatively lower detection rate of psychological distress in our study can be explained by the fact that patients experience significantly serious stigma after confirming the diagnosis of advanced lung cancer (Maguire et al., 2019), which has an impact on the tendency of patients to deliberately conceal their psychological distress. Moreover, as stated in our previous study (Lei et al., 2021), DT is not specific to patients with cancer (Hong et al., 2015) and cannot differentiate the risk of initial psychological distress from the accumulated risk of psychological distress, which may be an explanation for our findings.

Leventhal’s commonsense model of illness representations proposes that individuals’ illness perceptions are the major determinants of their health outcomes (Diefenbach and Leventhal, 1996). The individual will concurrently construct or elaborate both cognitive and emotional representations of their symptoms and illness to relieve the adverse impacts resulting from the symptom or an illness (Dempster et al., 2011). Some studies have revealed the association between illness perception and psychological distress among different populations (Zhang et al., 2017, 2018, 2020b). In this study, we first investigated the association between illness perception and psychological distress among patients with lung cancer and determined that illness perception was positively related to psychological distress.

As a positive psychological trait, mindfulness was found to be beneficial for improving adverse psychological outcomes through effective self-designed regulation and keeping positive emotional status (Ludwig and Kabat-Zinn, 2008). We have previously determined the direct association between mindfulness and psychological distress in patients with lung cancer; however, this specific association was not held in this study, which may be explained by the relatively mild severity of psychological distress compared with our finding (Lei et al., 2021). Moreover, we did not separately investigate the relationships of facets in mindfulness scale and psychological distress (Burger et al., 2021). Interestingly, we first determined the negative association between mindfulness and illness perception and revealed that mindfulness has an indirect impact on psychological distress through correcting negative illness perception. Illness perceptions refer to the attitudes, beliefs, and expectations of patients about symptoms or illnesses (Dalili and Bayazi, 2019), which are related to health information behavior practices and coping strategies (Katavic et al., 2016). Several studies have established that mindfulness-based interventions were associated with increased positive health perceptions and health behaviors (Roberts and Danoff-Burg, 2010), which provide theoretical support for our findings.

This study also revealed another novel finding that mindfulness indirectly influenced psychological distress among patients with lung cancer through the mediating effect of illness perception and perceived stress. As we introduced earlier, a higher negative perception of symptoms or illness represented worse psychological outcomes (Weinman et al., 1996). As one of the most common psychosocial risk factors, perceived stress has been suggested as a precursor state of adverse psychological outcomes because it has a negative impact on individual psychological adjustment (Kim and Jang, 2020). Previous studies also revealed the predictive effect of illness perception on perceived stress (Miceli et al., 2019; Sadeghi et al., 2019), which was further demonstrated in this study.

This study has some potential limitations that should be further interpreted. First, we used a convenience sample to investigate the associations between variables, which may introduce bias. Second, we calculated the theoretical sample size according to the number of variables, rather than performing an estimation based on acceptable statistical power. Third, all the patients were recruited from a single hospital in a single city and the sample size was relatively small; therefore, the generalizability of the study is questionable. Fourth, the level of mindfulness, illness perception, perceived street, and psychological distress were measured by using the self-report instruments, which may introduce subjective bias from patients. Definitively speaking, the prevalence of psychological distress and the type of scale could have some effects on the results.

## Conclusion

This study first shows that illness perception is positively related to the perceived stress and psychological distress in patients with lung cancer and negatively related to mindfulness. This study provided some evidence for the hypothesis that mindfulness can relieve the severity of psychological distress by decreasing the level of negative illness perception alone or decreasing the level of negative illness perception and perceived stress simultaneously. Based on these findings, illness perception and perceived stress screening should be enrolled in mindfulness-based intervention strategies for patients with lung cancer. Certainly, the total effect of mindfulness on psychological distress through the two targeted pathways in this study was very low; we, therefore, suggest continuing to explore other potential mechanisms.

## Clinical Implications

This study further clarified the potential mechanism of mindfulness on psychological distress through influencing illness perception and perceived stress in patients with lung cancer. From our current findings, mindfulness-based intervention protocol focusing on the correction of illness perception and reduction of perceived stress may be feasible and effective in improving psychological distress among patients with lung cancer.

## Data Availability Statement

The raw data supporting the conclusions of this article will be made available by the authors, without undue reservation.

## Ethics Statement

The studies involving human participants were reviewed and approved by the Institutional Review Board of Chongqing University Cancer Hospital. Written informed consent to participate in this study was provided by the participants’ legal guardian/next of kin.

## Author Contributions

LT and XT: had full access to all of the data in the study and are held responsible for the integrity of the data and accuracy of the data analysis. X-PQ, LT, XT, and MJ-H: concept and design. X-PQ, LT, XT, and L-JY: acquisition, analysis, or interpretation of data. X-PQ, XT, and MJ-H: drafting of the manuscript. XT, G-HC, and MJ-H: critical revision of the manuscript for important intellectual content. X-PQ and XT: statistical analysis. XT: obtaining funding, administrative, technical, or material support. MJ-H: supervision. All authors contributed to the article and approved the submitted version.

## Conflict of Interest

The authors declare that the research was conducted in the absence of any commercial or financial relationships that could be construed as a potential conflict of interest.

## Publisher’s Note

All claims expressed in this article are solely those of the authors and do not necessarily represent those of their affiliated organizations, or those of the publisher, the editors and the reviewers. Any product that may be evaluated in this article, or claim that may be made by its manufacturer, is not guaranteed or endorsed by the publisher.
